# Diverse Molecular Targets for Chalcones with Varied Bioactivities

**DOI:** 10.4172/2161-0444.1000291

**Published:** 2015-08-22

**Authors:** Bo Zhou, Chengguo Xing

**Affiliations:** Department of Medicinal Chemistry, College of Pharmacy, University of Minnesota, Minneapolis, USA

**Keywords:** Chalcone, Molecular target, Mechanism of action

## Abstract

Natural or synthetic chalcones with different substituents have revealed a variety of biological activities that may benefit human health. The underlying mechanisms of action, particularly with respect to the direct cellular targets and the modes of interaction with the targets, have not been rigorously characterized, which imposes challenges to structure-guided rational development of therapeutic agents or chemical probes with acceptable target-selectivity profile. This review summarizes literature evidence on chalcones’ direct molecular targets in the context of their biological activities.

## Introduction

**Figure 1:**
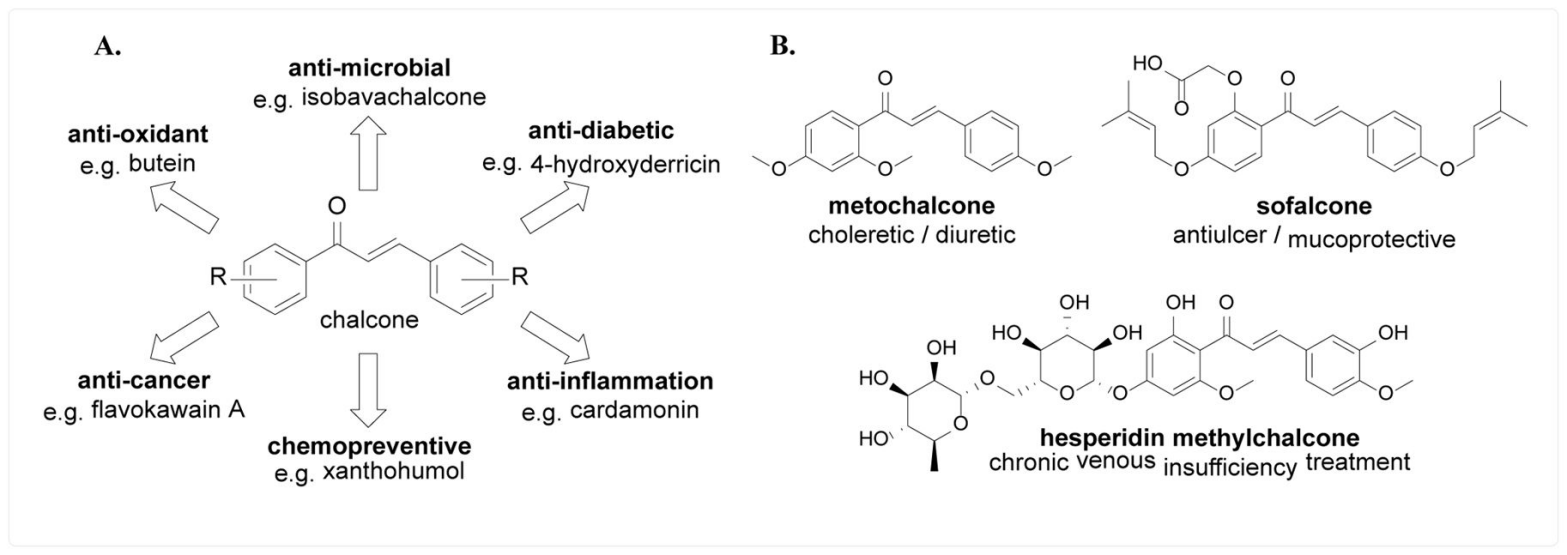
A. Structure scaffold of chalcone and examples of bioactivities; B. Examples of chalcones that have been marketed or clinically tested.

Chalcone or (E)-1,3-diphenyl-2-propene-1-one, is an important chemotype that has attracted great research interest for decades due to the high natural abundance of chalcone compounds, their easy synthesis, and most importantly, their diverse biological activities. Indeed, many natural chalcones demonstrated a wide variety of bioactivities, including anti-cancer, anti-inflammatory, anti-diabetic, cancer chemopreventive, anti-oxidant, and anti-microbial activities (Figure 1A) [1–3]. In many cases, a single compound exhibits several bioactivities. For example, xanthohumol revealed anti-cancer, cancer chemopreventive, anti-oxidant, and anti-inflammatory activities; isoliquiritigenin showed anti-inflammatory, anxiolytic, and anti-pigmentation activities; and isobavachalcone demonstrated chemopreventive, anti-cancer, anti-bacterial and anti-fungal activities [1]. Several chalcone compounds have been marketed or clinically tested for various health conditions (e.g., metochalcone – choleretic/diuretic; sofalcone – anti-ulcer/mucoprotective; and hesperidin methylchalcone – vascular protective), exemplifying the clinical potential of chalcones (Figure 1B) [2].

The wide bioactivity spectrum of chalcones, on the other hand, also indicates a potentially promiscuous target profile, which presents a challenge for their clinical development. Therefore, knowledge of chalcones’ mechanisms and direct molecular targets is particularly important for the future development of clinically useful chalcone compounds. A great number of reports have attempted to reveal mechanisms responsible for chalcones’ various bioactivities. Some of such mechanistic knowledge has been extensively reviewed, including anti-cancer [3–9], anti-inflammatory [9–11], and anti-diabetic activities [12], which will not be the focus of this review. Similarly, many potential direct molecular targets have been identified, yet have not been systematically reviewed. This review, therefore, summarizes the current knowledge of potential molecular targets that chalcones directly interact with. Various levels of experimental evidence of direct interaction and the biological relevance of these interactions is discussed. Due to the scope of this review, compounds derived from chalcone but do not contain the intact chalcone scaffold are not included.

## Molecular Targets of Chalcones

### Microtubule

Microtubule is the key component of mitotic spindles during cell proliferation. It is formed by heterodimers of α- and β-tubulin through a highly dynamic polymerization-depolymerization process. Anti-microtubule agents, which interact with microtubule and disturb such a dynamic process, typically cause cell cycle arrest. Anti-microtubule agents therefore are potential anticancer drugs; indeed paclitaxel, vinblastine, ixabepilone, and other anti-microtubule agents have been used clinically to treat a variety of malignancies [13,14].

**Figure 2:**
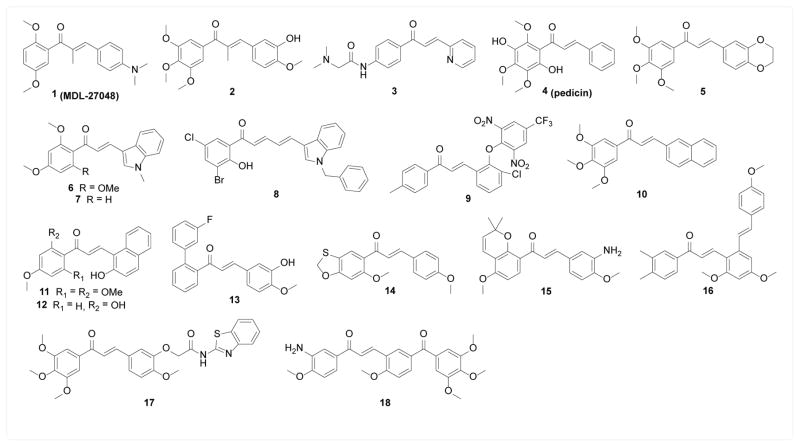
Structures of anti-microtubule chalcones.

A number of chalcone compounds have been shown to be potential anti-microtubule agents. The earliest example is compound 1 (MDL-27048). Peyrot et al. [15,16] found that this synthetic cytotoxic compound rapidly and reversibly bound to purified tubulin, inhibited tubulin polymerization into microtubule, and disrupted microtubule structure and arrested mitosis *in vitro*. Taking advantage of the change in spectroscopic properties (absorption and fluorescence) upon tubulin binding, 1 has been characterized as a single-site ligand of tubulin with a binding affinity of 2.8 × 10^6^ M^−1^. Classical colchicine binding site ligands podophyllotoxin and MTC competed with 1 for tubulin binding, suggesting that colchicine binding site was involved in the binding interaction. Silence et al. [17] used subsite-specific ligands to compete with 1 for tubulin binding and concluded that A-subsite but not C-subsite of colchicine binding-site was occupied by 1. In combination with other colchicine binding-site anti-microtubule agents, Ducki et al. [18] performed a 5D-QSAR study and concluded that the methyl substitution on the α-alkene position induced a significant change in the preferred conformation from s-cis to s-trans that favored tubulin binding, which potentially explained the higher potency observed with α-methyl chalcones. Similarly, α-methyl chalcone compound 2, designed by Ducki et al. [19], exhibited anti-microtubule activity biochemically and potent cytotoxicity *in vitro* through disrupting microtubule structure and inducing cell cycle arrest. Kim et al. [20] modeled the binding interaction of 1 with tubulin and found an adjacent unoccupied pocket, based on which compound 3 was designed. 3 exhibited micromolar potency for inhibiting tubulin polymerization and cancer cell growth but without significant improvement in potency. Alias et al. [21] isolated a cytotoxic chalcone 4 (pedicin) from *Fissistigma lanuginosum*, which inhibited tubulin polymerization biochemically with an IC50 of 300 μM. Lawrance et al. [22] synthesized six chalcones targeting anti-microtubule activity, four of which showed micromolar anti-microtubule potency in biochemical assays and sub-micromolar cytotoxicity against a panel of cancer cell lines with compound 5 being the most potent. Boumendjel et al. [23] and Martel-Frachet et al. [24] synthesized two closely related cytotoxic chalcones containing an indole moiety, 6 and 7, both of which inhibited tubulin polymerization biochemically and demonstrated *in vivo* anticancer activity in mouse xenograft models. In addition, 7 was able to compete off tubulin-bound colchicine, suggesting the involvement of colchicine-binding site in the binding interaction. Dyrager et al. [25] synthesized a series of dihalogenated chalcones and related dienones, and found compound 8 to be a microtubule stabilizer which fell in the same category as paclitaxel. Similarly, chalcones 9–14 were developed as anti-microtubule agents and showed cytotoxicity against tumor cell lines via cell cycle arrest [26–31]. In addition, compound 10 inhibited tumor cell migration [27], another microtubule-related activity, and compound 12–13 demonstrated *in vivo* antitumor activity in xenograft models [28,30]. Other anticancer pharmacophores have also been fused with the chalcone scaffold and yielded several novel anti-microtubule agents. Wang et al. [32,33] and Yang et al. [34] designed and synthesized a series of chalcones fused with a pyran ring to mimic cytotoxic natural product millepachine, among which compound 15 showed the best cytotoxicity towards a panel of cancer cells. Ruan et al. [35] designed compound 16 by incorporating a resveratrol moiety into chalcone scaffold, and Kamal et al. [36,37] designed compound 17 and 18 by incorporating either an amidobenzothiazole or a phenstatin moiety into chalcone core. All of these compounds were shown to be anti-microtubule agents that exhibited cytotoxicity against various cancer cell lines (Figure 2).

### Kinases

Protein phosphorylation, catalyzed by over 500 kinases encoded by human genome, regulates most if not all aspects of cell life. Dysregulation of kinase activities is associated with a variety of disorders including cancer, inflammatory diseases, diabetes, infectious diseases, and cardiovascular diseases. Kinase inhibitors as potential therapeutics have thus attracted great research attention for decades, with more than 30 clinically approved drugs to date, and many more in clinical trials [38–41]. Numerous literature reports have shown the potential of chalcones to regulate kinase activities through either direct enzymatic inhibition or altering kinase expression. Since this review focuses on chalcone’s direct targets, we will only discuss those examples that reveal direct kinase inhibition.

#### IKKs

**Figure 3:**
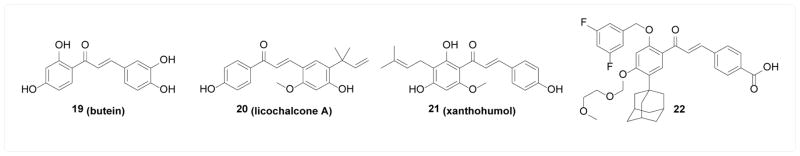
Structures of chalcones as IKK inhibitors.

IκB kinases (IKKs) are key regulators of the NF-κB signaling pathway, which plays an important role in cell response to various stimuli such as TNF, IL-1, UV radiation, stress, and pathogenic assaults. The activation of IKKs leads to phosphorylation and degradation of IκB, and subsequently nuclear translocation of NF-κB that initiates downstream transcription of target genes. Inhibiting IKKs is therefore considered a promising approach for intervening NF-κB related health conditions, especially cancer and inflammatory diseases [42,43]. Pandey et al. [44] found that anticancer and anti-inflammatory natural chalcone compound 19 (butein) directly inhibited IKKβ activity both biochemically and in cells, and subsequently reduced the downstream products of NF-κB activation, resulting in elevated apoptosis induced by TNF and other chemotherapeutic agents. In addition, cysteine 179 in IKKβ was found to be crucial to this inhibition, suggesting that a covalent Michael-type interaction of 19 with IKKβ at this residue might be involved. Similar observations were made by Funakoshi-Tago et al. [45] and Harikumar et al. [46], where 20 (licochalcone A) and 21 (xanthohumol) directly inhibited IKKβ through the involvement of cysteine 179 residue as well. Synthetically, series of adamantyl chalcones were developed by Bayon et al. [47], Lorenzo et al. [48,49] and Garcia-Rodriguez et al. [50] as cytotoxic agents; many of them were found to inhibit IKKα and IKKβ both biochemically and in cells and the inhibitory activity correlated well with the *in vitro* cytotoxicity. Compound 22 was the most potent inhibitor among this series with low micromolar potency (Figure 3).

#### Aurora kinases

**Figure 4:**
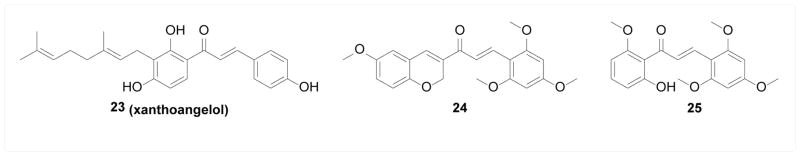
Structures of chalcones as Aurora kinase inhibitors.

Aurora kinases are key regulators of mitosis whose aberrant expression is found in various types of cancer. Aurora A phosphorylates Polo like kinase 1 (PLK1) which then phosphorylates Cdc25C and Wee1 and subsequently activates cyclin B-CDK1 complexes to promote mitotic entry. Aurora B is critical for correct microtubule-kinetochore attachments, the establishment of the spindle assembly checkpoint and cytokinesis. Both of them are therefore promising anticancer targets [51]. Limper et al. [52] studied natural products from *Psoralea corilifolia* and a geranyl chalcone 23 (xanthoangelol) was identified to inhibit both Aurora A and B kinases with micromolar potency and induce apoptotic cell death in cancer cell lines. Shin et al. [53] synthesized a library of chromenyl chalcones. Compound 24, demonstrating the most potent cytotoxicity against cancer cell lines and inhibition of colony formation, inhibited both Aurora A and B kinases with no effect on other kinases. Shin et al. [54] also synthesized a series of chalcones as cytotoxic compounds against ovarian cancer cells, where the most potent compound 25 was found to be an Aurora A inhibitor (Figure 4).

#### Receptor tyrosine kinases

**Figure 5:**
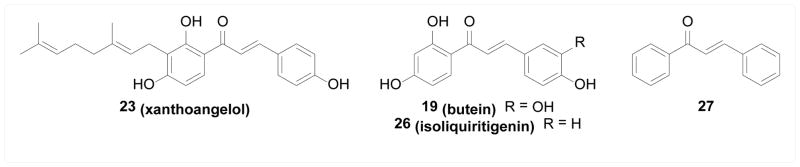
Structures of chalcones as inhibitors of RTKs.

Receptor tyrosine kinases (RTKs), such as VEGFR, EGFR, and FGFR, are cell signaling effectors that regulate normal development and homeostasis, whose aberrant activities are responsible for many pathological conditions, especially cancers. Many small molecule inhibitors of RTKs are clinical cancer therapies [55,56]. Aforementioned study by Limper et al. [52] found that the natural geranyl chalcone 23 (xanthoangelol) inhibited not only the Aurora kinases but also EGFR activity in enzymatic assays, which may contribute to its cytotoxicity. Yang et al. [57,58] showed that 19 (butein) was a micromolar inhibitor of EGFR and that this inhibition was competitive against ATP and non-competitive against the substrate, suggesting the ATP binding site being the potential binding pocket. Jung et al. [59] showed that the cytotoxic natural chalcone 26 (isoliquiritigenin) inhibited not only wild-type EGFR, but also a mutant type prevalent in non-small-cell lung cancer cells, in an ATP-competitive manner. Xenograft study revealed *in vivo* anticancer efficacy of isoliquiritigenin against cancer cells expressing mutant EGFR. The same compound was studied by Wang et al. [60] and inhibited VEGFR2 enzymatic activity and consequently inhibited endothelial cell growth and angiogenesis induced by VEGF *in vivo*. Lee et al. [61] found that the simple unsubstituted chalcone 27 inhibited VEGFR biochemically as well as tumor growth and angiogenesis in a xenograft model. Interestingly, many other RTKs besides VEGFR were also found to be inhibited by 27, such as EphB2, FGFR3, and MSPR (Figure 5).

#### Other kinases

**Figure 6:**
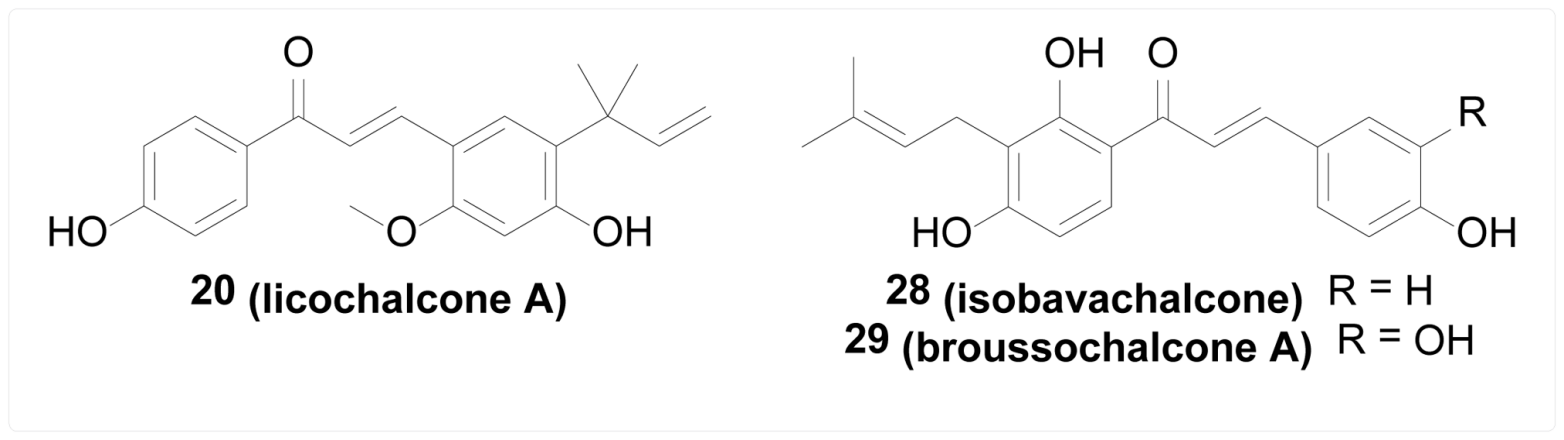
Structures of chalcones as inhibitors of other kinases.

Song et al. [62] studied the anti-inflammatory natural chalcone 20 (licochalcone A) and found that it suppressed UV-induced COX-2 expression, an important proinflammatory enzyme, through direct binding and inhibition of kinases PI3K, MEK1, and B-Raf. The binding study was performed with bead-immobilized compound and revealed that ATP competed the binding of 20 to PI3K and B-Raf, but not MEK1, suggesting different binding sites/modes with different kinases. Jing et al. [63] studied the cytotoxicity of natural chalcone compound 28 (isobavachalcone) against various cancer cell lines, which was potentially attributed to its inhibitory activity against Akt, a key effector in the PI3K/Akt signaling pathway regulating cell proliferation and growth and hence a promising anticancer target. Wang et al. [64] studied the mechanisms by which 29 (broussochalcone A) attenuated respiratory burst in stimulated neutrophils and protein kinase C (PKC) was found to be directly inhibited by broussochalcone A, and the interaction was shown to be at the catalytic domains (Figure 6).

### Oxidoreductases

#### Cyclooxygenase and 5-lipoxygenase

**Figure 7:**
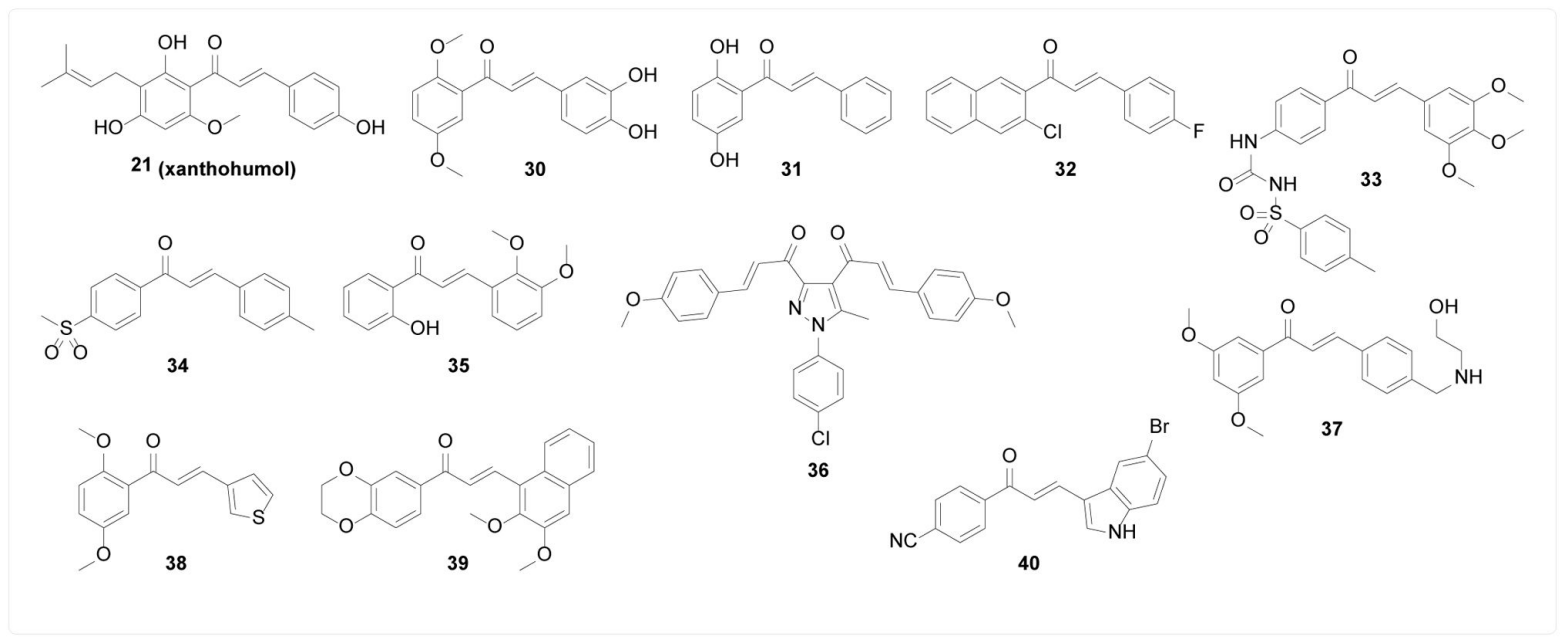
Structures of chalcones as COX inhibitors.

Cyclooxygenase (COX) and 5-lipoxygenase (5-LOX) are key enzymes in the arachidonic acid (AA) metabolic pathway, whose end products are inflammatory mediators such as prostaglandins (PGs), leukotrienes (LTs), and thromboxanes (TXs) that are highly involved in the pathology of various inflammatory diseases and cancers. Therefore, COX and 5-LOX have long been considered potential therapeutic targets for these diseases [65,66]. The chalcone scaffold has been widely explored as a pharmacophore to inhibit COX and 5-LOX. Gerhauser et al. [67] found that natural chalcone 21 (xanthohumol) inhibited both isotypes of COX with higher potency against COX-1 than COX-2; however, no further biological relevance was reported. Sogawa et al. [68] synthesized a series of chalcones bearing 3,4-dihydroxy functional groups and many of them were potent 5-LOX inhibitors with nanomolar potency and moderate COX inhibitors with micromolar potency. Further *in vivo* anti-inflammation efficacy was evaluated using AA-induced mouse ear edema model, and the most potent compound 30 was among the most potent inhibitors of both enzymes in their library. Lin et al. [68] studied several natural chalcone products for their inhibitory activity against AA-induced platelet aggregation and identified 29 (broussochalcone A) as the most potent candidate, which was explained by its COX inhibitory activity. Lin et al. [69] further expanded the chalcone library as well as the anti-inflammatory scope, where compound 31 was identified as a COX inhibitor that potently inhibited AA- or adrenaline-induced platelet aggregation, compound 48/80-induced mast cell degranulation, and peptide fMLP-induced neutrophil activation. Herencia et al. [71] synthesized a series of 2-chloroquinolinyl chalcones and compound 32 was found to inhibit both 5-LOX and COX. *In vivo* study showed that it reduced the production of PGE2 and LTB4 by stimulated leukocytes. Araico et al. [72] developed a series of phenylsulphonyl urenyl chalcones as dual inhibitors of COX-2 and 5-LOX, and the most potent inhibitor 33 demonstrated potent reduction in PGE2 and LTB4 production *in vitro*. Zarghi et al. [73] designed a series of chalcones bearing a p-methylsulphonyl group as it frequently appears in the structure of COX-2 inhibitors, and the most potent inhibitor 34 showed a more than 100-fold selectivity towards COX-2 than COX-1 without further biological evaluation. Tran et al. [74] studied the inhibitory effect of 2′-hydroxychalcones on LPS-induced cell-based PGE2 production and the most potent compound 35 was docked into COX-2 binding pocket without further biochemical confirmation. El-Sabbagh et al. [75] incorporated an N-arylpyrazole moiety, the major pharmacophore in celecoxib, into a bis-chalcone scaffold and obtained several selective COX-2 inhibitors with *in vivo* edema inhibition activity. Compound 36 was the most potent and selective inhibitor in this series, and its activity profile was comparable to that of celecoxib. Jantan et al. [76] studied synthetic chalcone derivatives and found compound 37 biochemically inhibited both isotypes of COX as well as 5-LOX. Wu et al. [77] studied several heterocyclic chalcones for their cell-based inhibitory activity against AA- and collagen-induced platelet aggregation and TXB2 formation, where the most potent compound 38 exhibited COX-1 enzymatic inhibition. Bukhari et al. [78] studied a series of naphthyl chalcone derivatives and found compound 39 as a non-selective COX inhibitor with micromolar potency. Finally, Ozdemir et al. [79] evaluated a series of indole-chalcones as COX inhibitors, among which the most potent inhibitor compound 40 showed *in vivo* anti-inflammation activity (Figure 7).

#### Tyrosinase

**Figure 8:**
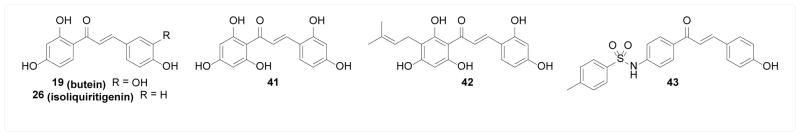
Structures of chalcones as tyrosinase inhibitors.

Tyrosinase is the primary enzyme catalyzing the multi-step oxidation of tyrosine in the production of melanin, the skin pigment. Increased tyrosinase activity can cause various types of hyperpigmentation disorders, hence an optimal drug target for such conditions [80] Nerya et al. [81] investigated the tyrosinase inhibitory activity of licorice-derived natural products and identified 26 (isoliquiritigenin) as a tyrosinase inhibitor both biochemically and in melanocytes. Further studies by Nerya et al. [82] and Khatib et al. [83] identified 19 (butein) and a few other chalcones as tyrosinase inhibitors with comparable potency. Interestingly, 19 itself was found to be a substrate of tyrosinase and the o-quinone product was detected with LC-MS. Jun et al. [84] synthesized a series of 2′,4′,6′-trihydroxychalcones, among which compound 41 was the most potent tyrosinase inhibitor. Kinetic study revealed competitive inhibition of 41 against tyrosine as the substrate, giving insight into the potential binding site. Zhang et al. [85] studied the inhibitory effect of natural chalcone 42 on tyrosinase activity and melanin biosynthesis, and found that it competitively inhibited tyrosinase activity both biochemically and in cells. Seo et al. [86] evaluated several synthetic sulfonylamino chalcones for their anti-pigmentary effect biochemically and in cells, where compound 43 was demonstrated as the most potent inhibitor of melanin formation in cell-based experiment, although it wasn’t the most potent in enzymatic assay. Part of its cellular activity may be attributed to reduced protein level of tyrosinase as revealed by Western analysis. Finally, compound 43 was demonstrated to inhibit UV-induced skin pigmentation in brown guinea pigs (Figure 8).

#### Sex hormone converting enzymes

Sex hormones as important signaling messengers play major roles in the functions of reproductive organs, and their dysregulation is highly associated with diseases such as breast cancer and prostate cancer [87,88]. Enzymes involved in the biosynthesis of sex hormones are thus potential drug targets for such diseases. Chalcones have shown the potential to inhibit some of these enzymes, such as aromatase involved in estrogen synthesis, and 5α-reductase and 17β-hydroxysteroid dehydrogenase involved in androgen synthesis.

#### Aromatase

Wang et al. [89] screened five chalcones for aromatase inhibitory activity both biochemically and in MCF-7aro breast cancer cells. The most potent inhibitor 19 (butein) suppressed testosterone-induced MCF-7aro cell growth, which was synergistically enhanced by co-treatment with estrogen receptor antagonist ICI-182780. Similarly, natural chalcone 21 (xanthohumol) was identified by Monteiro et al. [90] as an aromatase inhibitor in a cell-based assay, and its anti-proliferative effect was reverted by addition of estradiol. Le Bail et al. [91] evaluated a series of natural and synthetic chalcones for aromatase inhibitory activity in biochemical assays, from which 44 (naringenin chalcone) was found to be the most potent inhibitor. Lee et al. [92] isolated twenty-two natural products from *Broussonetia papyrifera* and several potent aromatase inhibitors were identified with compound 45 of sub-micromolar potency.

#### 5α-Reductase

Shimizu et al. [93] identified geranyl chalcone 46 as a natural inhibitor of 5α-reductase biochemically, where the geranyl group was found to be important for potency. Similarly, Hussein et al. [94] isolated a β-hydroxyl chalcone 47 as a natural inhibitor of 5α-reductase in a cell-based assay.

#### 17β-Hydroxysteroid dehydrogenase

**Figure 9:**
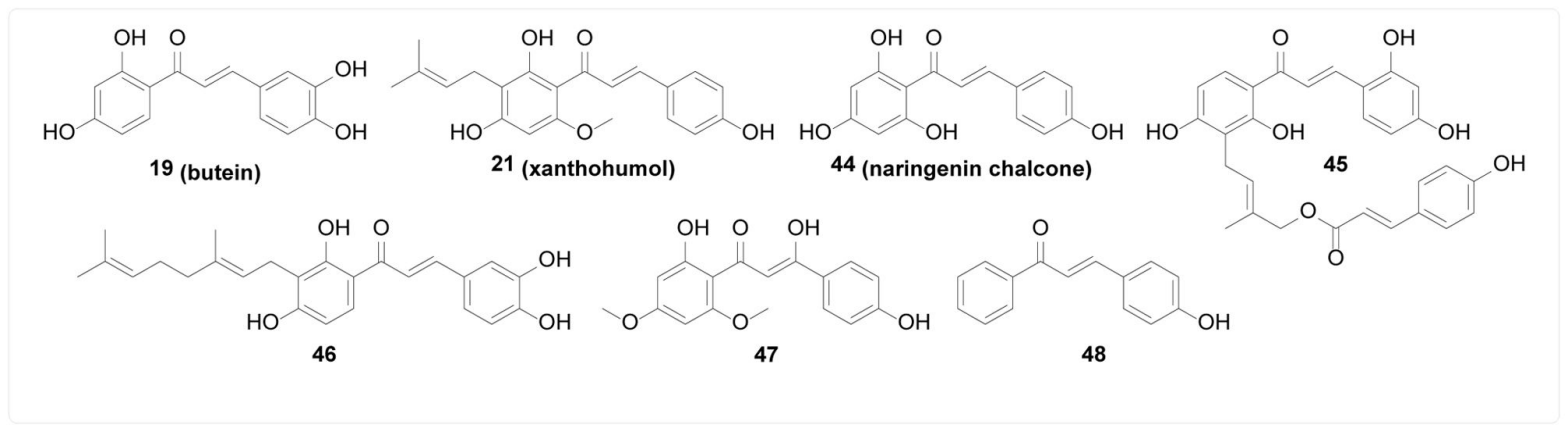
Structures of chalcones as inhibitors of sex hormone converting enzymes.

In the study by Le Bail et al. [91] mentioned above, in addition to aromatase inhibition, several chalcones also exhibited moderate inhibitory activity against 17β-hydroxysteroid dehydrogenase biochemically, among which 48 was the most potent. Cellular evidence of such interaction, however, is unavailable (Figure 9).

#### Aldose reductase

**Figure 10:**
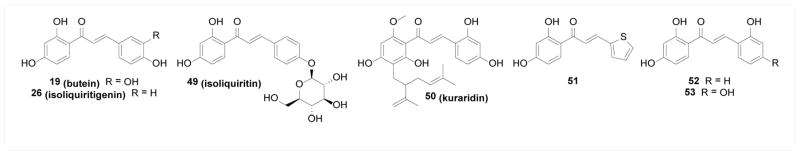
Structures of chalcones as ALR2 inhibitors.

Aldose reductase (ALR2) catalyzes the conversion of glucose to sorbitol, the first step in polyol pathway of glucose metabolism. Increased flux of glucose through polyol pathway under conditions such as diabetes initiates a complex cascade leading to oxidative stress and inflammation, which renders ALR2 a promising drug target for intervention of inflammatory conditions, particularly the chronic complications associated with diabetes [95,96]. Investigation of chalcone compounds as ALR2 inhibitors started with the discovery by Aida et al. [97,98] that natural chalcones 26 (isoliquiritigenin) and 49 (isoliquiritin) potently inhibited ALR2 activity biochemically. *In vivo* study showed that 26 significantly inhibited sorbitol accumulation in red blood cells, sciatic nerves, and lenses in diabetic rats. Similarly, Jung et al. [99] isolated natural chalcone 50 (kuraridin) from *Sophora flavescens* which exhibited ALR2 inhibitory activity biochemically. Lim et al. [100] studied a panel of chalcones and found 19 (butein), an equally potent ALR2 inhibitor as 26 in enzymatic assays, exhibited better inhibitory activity against sorbitol accumulation *in vivo*. Rastelli et al. [101] modeled the interaction between 26 and ALR2, and studied the structural requirements, where they found each of the three hydroxyl groups to be essential. Severi et al. [102] and Lim et al. [103] synthesized analogs of 26 and obtained equally potent inhibitors 51 and 52 without further biological investigation. By introducing an additional hydroxyl group onto 26, Iwata et al. [104] obtained nanomolar inhibitor 53 which is about 100-fold more potent than 26 (Figure 10).

#### Thioredoxin reductase

Thioredoxin system, consisting of thioredoxin reductase (TrxR), its substrate thioredoxin (Trx), and cofactor NADPH, is one of the major biological antioxidant systems and regulates various cellular processes. Overexpression of TrxR has been observed in many tumors, rendering it a potential target for chemotherapy [105,106]. Gan et al. [107] developed a panel of Michael acceptor-type pharmacophores for TrxR inhibition, among which chalcones (such as 54) and closely related 1,5-diphenyl-pent-1-en-3-ones (DPPen) proved to inhibit cellular TrxR activity. The good correlation between TrxR inhibitory potency and cytotoxicity within their library indicated involvement of TrxR inhibition in their cytotoxic mechanisms. Interestingly, the covalent adduct of the most potent DPPen analog with TrxR at selenocysteine residue U498 was observed via MS analysis, giving insight into potential interaction modes of chalcones with TrxR. Zhang et al. [108] screened the cytotoxicity of forty-four synthetic chalcones against a panel of cancer cell lines, and the most potent compound 55 was tested for TrxR inhibition. Its inhibition of TrxR was selective over other oxidoreductases such as glutathione reductase and glutathione peroxidase, and U498 residue was involved in the interaction as much weaker inhibition was observed with U498C mutant. In cells, 55 significantly decreased cellular thiol level and increased ROS level as a result of TrxR inhibition. The cytotoxicity of 55 was attenuated by antioxidant N-acetylcysteine, and enhanced by GSH synthesis inhibitor BSO, further supporting the important role of TrxR inhibition in the cytotoxic mechanism of 55. However, this could also be due to non-specific Michael addition with thiols, which deserves further clarification.

#### Monoamine oxidase

**Figure 11:**
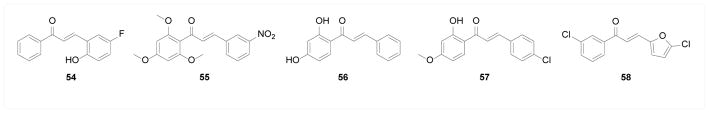
Structures of chalcones as TrxR and MAO inhibitors.

Monoamine oxidases (MAOs) are important metabolic enzymes catalyzing the oxidative degradation of monoamine neurotransmitters. Elevated activities of MAOs can lead to reduced cognitive ability and ROS accumulation, and is therefore associated with depression and neurodegenerative diseases, rendering MAOs important drug targets for such conditions [109]. Early study with natural products from *Glycyrrhiza uralensis* root by Tanaka et al. [110] identified several chalcones, such as compound 56, as competitive inhibitors of mitochondrial MAO from rat liver. Chimenti et al. [111] screened sixteen synthetic chalcones against MAO-A and MAO-B and most compounds showed inhibitory activity against MAO-B but not MAO-A, with potency ranging from low nanomolar to micromolar. The most potent compound 57 was four times more potent than clinically used MAO-B inhibitor selegiline and over 10,000-fold selective over MAO-A. The inhibition was irreversible as removing compound from enzyme did not result in recovery of enzymatic activity, suggesting potential covalent modification. Robinson et al. [112] synthesized a panel of furano-chalcones and they all showed competitive inhibition of MAO-B with micromolar potency but very weak or no inhibition of MAO-A. Interestingly, inhibition by the most potent compound 58 was reversible, contrary to the previous study. However, no further study was performed to investigate the potential contribution of MAO inhibition to chalcones’ bioactivities (Figure 11).

### Hydrolases

#### Proteases

##### Cathepsins

Cathepsins are cysteine proteases mainly involved in the bulk protein degradation within lysosomes. Recent findings have shed light on their involvement in many pathological processes, including cancer, arthritis, and atherosclerosis. High cathepsins activity is associated with cancer progression, invasion, and metathesis, thus making them potential drug targets [113]. Kim et al. [114] evaluated a panel of synthetic chalcones for cytotoxicity and cathepsin inhibition; the most cytotoxic compound 59 was the most potent inhibitor of cathepsin B and L with micromolar potency. Similarly, Ramalho et al. [115] screened fifteen synthetic chalcones and cytotoxic compound 60 was found to inhibit cathepsin K while none of the compounds significantly inhibited cathepsin B. Raghav et al. [116] synthesized twenty-seven flavonoids and screened them for cathepsin B and H inhibitory activity, where 2′-hydroxychalcones showed better potency than flavones and flavanones bearing the same substituents, and 4-nitro substitution was found to possess the best potency. Compound 61 with both structural features was the most potent inhibitor within their library. The inhibition kinetics was also studied and revealed a competitive mode of inhibition, suggesting potential binding at the catalytic site.

##### β-Secretase

**Figure 12:**
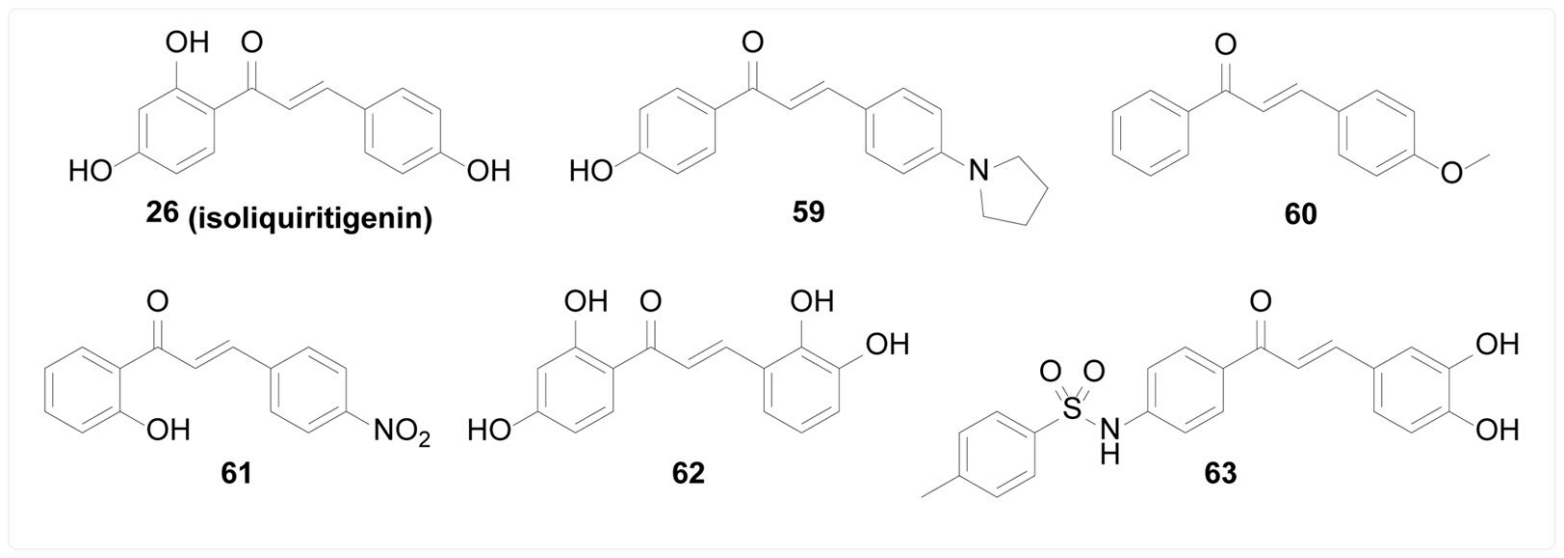
Structures of chalcones as inhibitors of proteases.

β-Secretase (BACE1) catalyzes the cleavage of β-amyloid precursor protein to produce neurotoxic peptide β-amyloid, whose aggregation is an important pathological hallmark for Alzheimer’s disease (AD). Therefore, inhibiting BACE1 has become an attractive anti-AD strategy [117]. Ma et al. [118] screened natural products from cognition enhancing herb *Glycyrrhiza uralensis* for BACE1 inhibitory activity and identified 26 (isoliquiritigenin) as a moderate BACE1 inhibitor. In an attempt to improve potency, a 22-membered library was constructed, from which compound 62 was identified as the most potent inhibitor with sub-micromolar potency. Similar potency was also achieve by Kang et al. [119], where compound 63 was identified from a series of sulfonamide chalcones as the most potent BACE1 inhibitor, with a reversible and mixed-type mode of inhibition. No further biological investigation was performed (Figure 12).

#### Esterase

##### Histone deacetylases

**Figure 13:**
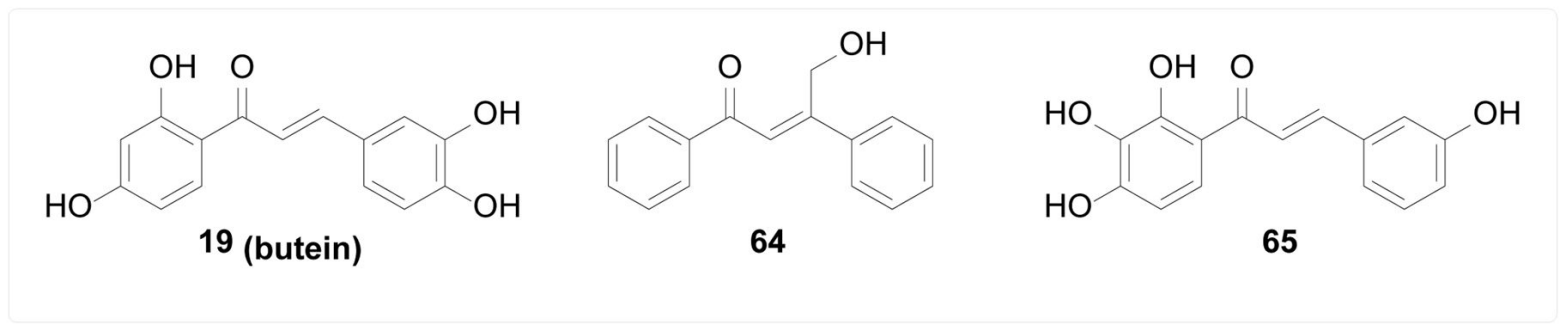
Structures of chalcones as HDAC inhibitors.

Histone deacetylases (HDACs) are epigenetic enzymes catalyzing the deacetylation of histone, which regulates the transcription of various genes. Aberrant expression/function of HDAC isoforms are involved in the pathology of many diseases, especially cancer, neurological diseases, and immune disorders [120, 121]. HDAC inhibitors and especially isoform-specific inhibitors are therefore potential drug candidates for these diseases. Orlikova et al. [122] screened twenty-one natural chalcones for HDAC inhibitory activity. Only a few compounds showed moderate inhibitory activity against total cellular HDACs with 19 (butein) being the most potent. Zhou et al. [123] employed computational strategies based on structures and mechanisms of different isoforms of HDACs to design selective HDAC2 inhibitors, and identified β-hydroxymethyl chalcone 64 as a selective HDAC2 inhibitor over HDAC1 and HDAC3. Kahyo et al. [124] screened a panel of flavonoids for inhibition of the deacetylase activity of SIRT1, a class III HDAC whose substrates include tumor suppressor protein p53, thus a potential anticancer target. Chalcone 65 was identified to inhibit SIRT1 and as a consequence increase the acetylated p53 level in cells and inhibit cell proliferation (Figure 13).

##### Cholinesterases

**Figure 14:**
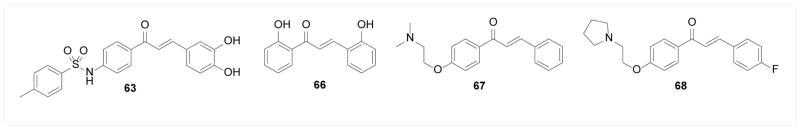
Structures of chalcones as AChE/BChE inhibitors.

Cholinesterases, including AChE and BChE, are esterases catalyzing the hydrolytic breakdown of neurotransmitter acetylcholine, and are hence involved in various neurological disorders, especially AD. Inhibitors of cholinesterases have been widely used in clinic for symptomatic treatment of AD [125,126]. The study performed by Kang et al. [119] as mentioned above with sulfonamide chalcones identified compound 63 not only as a potent BACE1 inhibitor, but also a moderate AChE/BChE inhibitor, further indicating its potential anti-AD effect. Ansari et al. [127] identified compound 66 from twenty-four synthetic chalcones as an AChE/BChE inhibitor with micromolar potency. Starting from 4-hydroxychalcone and guided by computational docking, Liu et al. [128] installed a tertiary amine substituent to form a cation-π interaction with tryptophan 84 residue and obtained compound 67 which was >100-fold more potent in AChE inhibition than the parent compound and also inhibited BChE. Kinetic study revealed it as a mixed-type inhibitor of both enzymes. Further study by Liu et al. [129] suggested that introduction of a fluorine atom resulted in enhanced potency, where the most potent compound 68 was 20-fold more potent than 67. However, no further biological study was performed (Figure 14).

##### Protein tyrosine phosphatases

**Figure 15:**
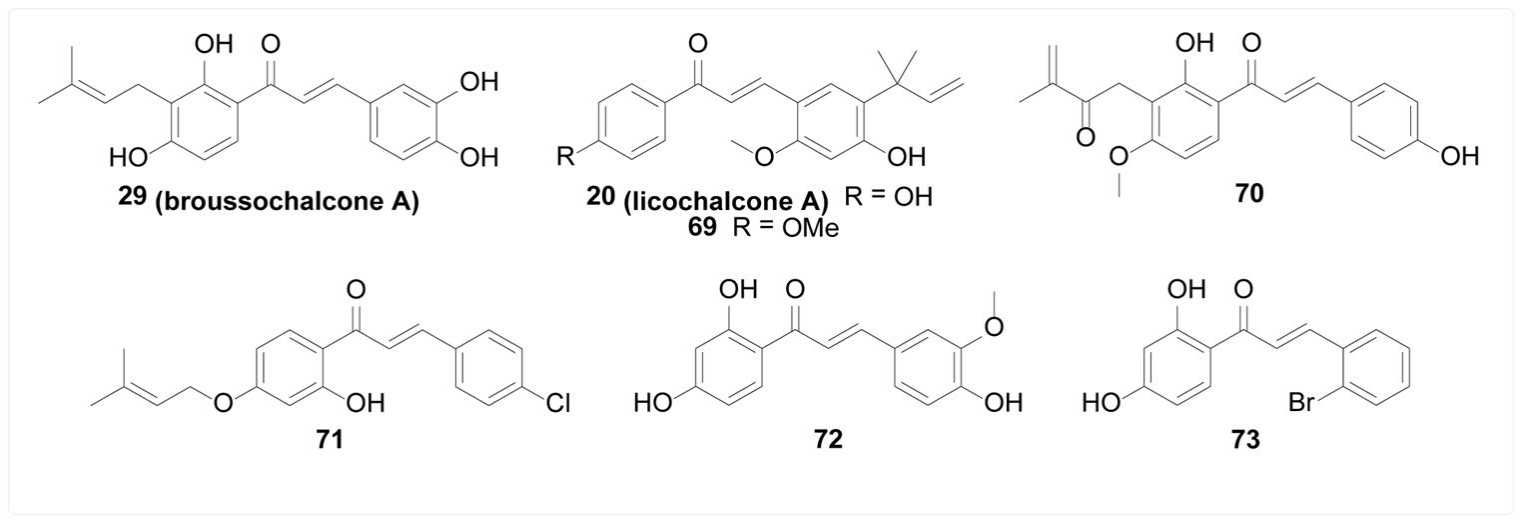
Structures of chalcones as PTP inhibitors.

Protein tyrosine phosphatases (PTPs) catalyze the hydrolytic dephosphorylation of phosphotyrosine residues of various protein substrates. Chalcones have shown potential interaction with at least two members of this enzyme family, namely PTP1B and CDC25B. PTP1B dephosphorylates and thereby inactivates insulin receptor kinase, and as a result elevated PTP1B activity is highly associated with decreased insulin sensitivity leading to diabetes and obesity [130]. CDC25B, on the other hand, is a mitotic regulator by dephosphorylating cyclin dependent kinases (CDKs) whose activity is essential for the G2/M transition in human cells, and thus a potential anticancer target [131]. Chen et al. [132] identified five natural products from *Broussonetia papyrifera*, including 29 (broussochalcone A), as moderate PTP1B inhibitors. Similarly, Yoon et al. [133] identified 21 (licochalcone A) from *Glycyrrhiza inflata* as a moderate PTP1B inhibitor, and through semisynthetic derivatization compound 69 was obtained with 2-fold improved potency than that of 21. Li et al. [134] isolated twenty-two natural products, ten of which are chalcones, from *Angelica keiskei* and screened them for PTP1B inhibitory activity, where compound 70 showed sub-micromolar potency of inhibition in a competitive manner. Zhang et al. [135] evaluated nineteen 2′-hydroxy-4′-isoprenyloxychalcones for CDC25B inhibition, where compound 71 was identified as the most potent analog. Sun et al. [136] synthesized twenty-one 2′,4′,6′-trihydroxychalcones and identified compound 72 as a sub-micromolar inhibitor of both PTP1B and CDC25B in a competitive manner. Similarly, Xie et al. [137] identified compound 73 from eleven 2′,4′-dihydroxychalcones as an inhibitor of both CDC25B and PTP1B. 73 also exhibited micromolar inhibitory activity against cancer cell proliferation as well as *in vivo* tumor growth inhibition, which might be associated with CDC25B inhibition (Figure 15).

##### Secreted phospholipase A2

**Figure 16:**
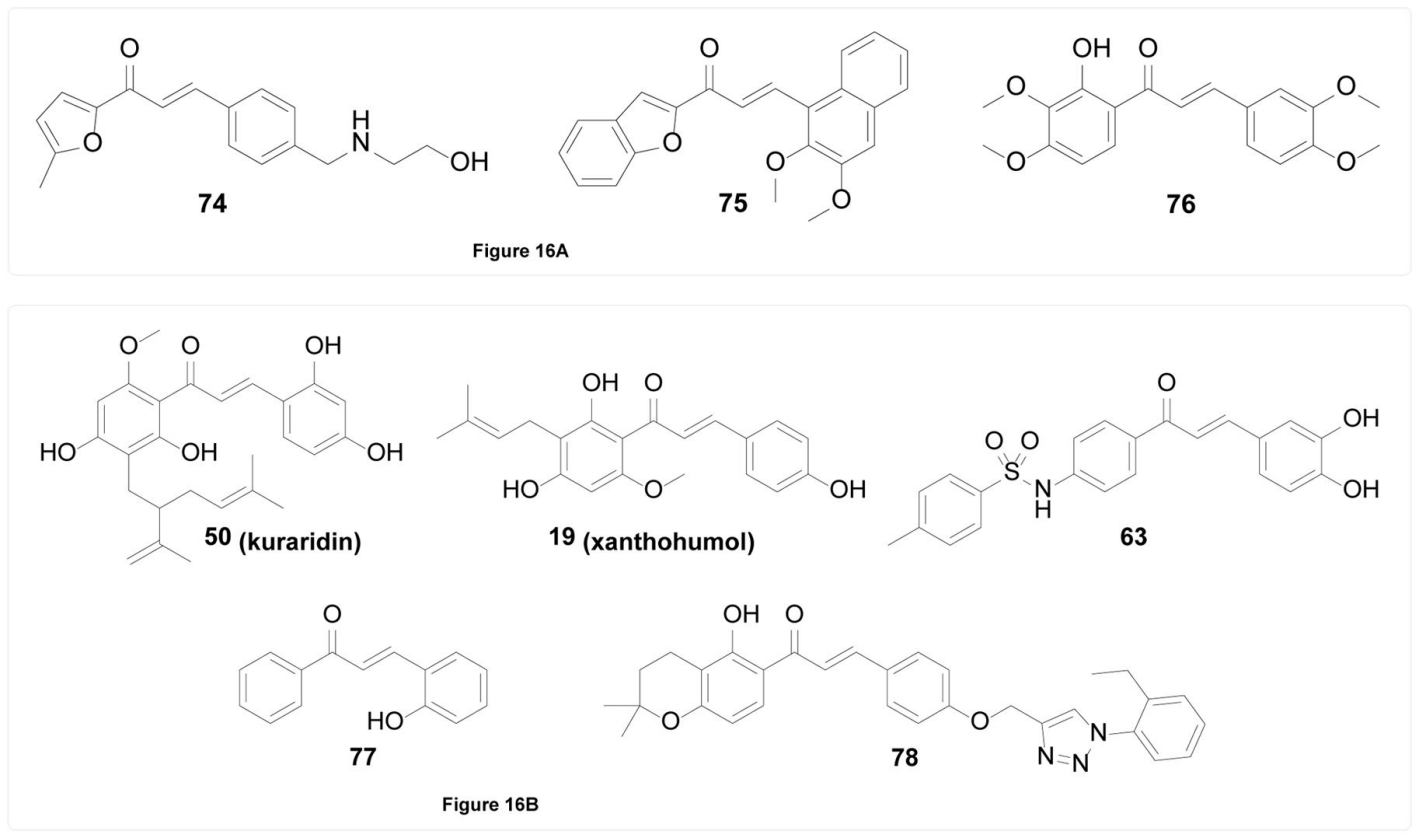
Figure 16A. Structures of chalcones as sPLA2 inhibitors. Figure 16B. Structures of chalcones as α-glucosidase inhibitors.

Secreted phospholipases A2 (sPLA2) are extracellular enzymes catalyzing the hydrolytic release of fatty acids, including inflammatory intermediate arachidonic acid. Given its important role in inflammation as the enzyme catalyzing the first step of the arachidonic pathway, sPLA2 has long been considered a promising target for treatment of many inflammation-related diseases [138]. Two studies mentioned above by Jantan et al. [76] and Bukhari et al. [78] identified not only synthetic chalcones as COX inhibitors, but also moderate sPLA2 inhibitors such as compounds 74 and 75. In addition, chalcone 76 was identified by Ballesteros et al. [139] as a sPLA2 inhibitor with cell-based anti-inflammatory activity where it inhibited LTB4, TXB2, and elastase production by stimulated neutrophils (Figure 16A).

##### α-Glucosidase

α-Glucosidase is an intestinal enzyme to release α-glucose from dietary polysaccharide for glucose absorption, and hence a valid anti-diabetic target on which commercial drugs such as acarbose and miglitol act [140]. Chalcones have been shown to possess potential inhibitory activity against α-glucosidase. Natural chalcone 19 (xanthohumol) was shown by Liu et al. [141] to exhibit moderate α-glucosidase inhibitory activity in a non-competitive manner. Interestingly, the interaction with xanthohumol resulted in quenched intrinsic fluorescence, reduced hydrophobicity, and changes in CD spectrum of α-glucosidase, all of which suggested a conformational change that may explain its non-competitive inhibition. Another natural chalcone 50 (kuraridin) was identified by Kim et al. [142] among a panel of flavonoids from *Sophora flavescens* as a moderate non-competitive inhibitor of α-glucosidase. Synthetically, Seo et al. [143] found that introduction of a sulfonamide substituent significantly increased chalcones’ inhibitory activity against α-glucosidase, and compound 63 which showed BACE1 and AChE/BChE inhibitory activity as mentioned above was also the most potent chalcone α-glucosidase inhibitor with sub-micromolar potency. Similarly, the study by Ansari et al. [127] mentioned above also identified chalcones as moderate inhibitors of α-glucosidase and 77 showed the best potency. Chinthala et al. [144] developed a series of chalcone triazoles as potential α-glucosidase inhibitors, but the most potent compound 78 only showed mid-micromolar potency (Figure 16B).

### Microbial enzymes for antimicrobial activity

**Figure 17:**
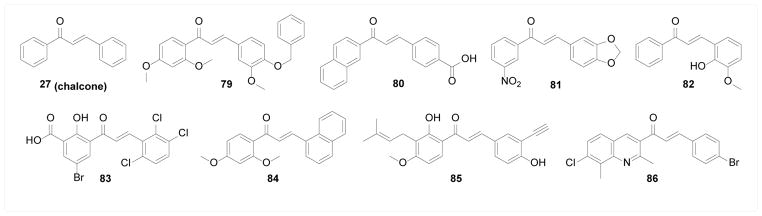
Structures of chalcone compounds targeting microbial enzymes.

Quite a number of chalcone compounds have shown antimicrobial activity against a spectrum of pathogens including bacteria, fungi, parasites, and viruses [1,3]. Various potential direct molecular targets have been suggested to account for such activities. Chiaradia et al. [145,146] and Mascarello et al. [147] discovered chalcone inhibitors of the bacterial enzymes MtbPtpA and MtbPtpB, which are protein tyrosine phosphatases secreted by *Mycobacterium tuberculosis* into host cells that attenuate host immune defenses. Compound 79 was identified as the most potent MtbPtpA inhibitor, whereas compound 80 was the most potent inhibitor of MtbPtpB. However, no data for their anti-tuberculosis activity is available. Another bacterial tyrosine phosphatase critical for Yersinia pathogenesis, YopH, was found to be inhibited by several synthetic chalcones [148] with compound 81 being the most potent inhibitor. Deng et al. [149] screened an NCI antiviral compound library for HIV-1 integrase inhibitory activity and identified compound 82 as the most potent inhibitor with *in vitro* anti-HIV activity. Sharma et al. [150] synthesized a chalcone library incorporating a 3-keto salicylic acid moiety targeting HIV-1 integrase, where compound 83 was identified as the most potent inhibitor with a higher *in vitro* anti-HIV potency than 82. Geyer et al. [151] discovered compound 84 as a potent inhibitor of Pfmrk, a cyclin-dependent kinase critical for the cell cycle of *Plasmodium falciparum* and thus an attractive target for malaria. However, the weak correlation between antimalarial activity and Pfmrk inhibition suggests the potential involvement of other mechanisms. Battenberg et al. [152] employed a chemical probe approach to study the antibacterial target of the natural product 4-hydroxyderricin. Probe 85 showed similar antibacterial activity as 4-hydroxyderricin. In subsequent target profiling experiment, it covalently pulled out seryl-tRNA synthase (STS) from the lysate of *S. aureus*, suggesting STS as the direct target of 85, which was confirmed by enzymatic inhibition assay. These results overall provided compelling evidence that STS is the direct target responsible for its antibacterial activity. Similarly, Wallock-Richards et al. [153] identified the simple unsubstituted chalcone 27 as a covalent inhibitor of Sortase A (SrtA), a bacterial transpeptidase involved in host cell attachment. The covalent labeling of SrtA by chalcone was revealed by mass spectrometry and confirmed with biochemical enzymatic inhibition assay. Abdullah et al. [154] introduced a quinoline moiety onto chalcone scaffold and identified compound 86 from a quinolone-chalcone library as the most potent inhibitor of bacterial DNA gyrase, which correlated well with its antibacterial activity tested against several strains of bacteria (Figure 17).

### Receptors

#### Retinoic acid receptors

Retinoic acid receptors (RARs) are nuclear receptors that function as ligand-dependent transcription factors and thereby play important roles in various physiological as well as pathological processes including cancer and inflammation [155]. Although much cell-based evidence exists in literature for chalcone’s potential effect on RAR activity, these results were typically obtained from classical luciferase-based assays which could be due to a combination of effects on transcription-regulated protein level changes or post-translational modifications, and will not be discussed given the scope of this review. Nevertheless, evidence does exist that chalcones may directly interact with RARs. Kagechika et al. [156] synthesized a panel of carboxyl-containing chalcones and flavonoids, among which 87 and 88 were identified as potential RAR agonists that exhibited retinoidal effect - inducing differentiation in HL60 cells. These compounds were later confirmed to directly bind to all three isoforms of RARs with sub-nanomolar affinity [157,158]. Importantly, Klaholz et al. [158] obtained the co-crystal of 88 with the ligand binding domain of RARγ, and the crystal structure clearly showed direct interaction of 88 with RARs in the ligand binding pocket.

#### Estrogen receptor

Estrogen receptors (ERs) are sex hormone receptors acting as transcription factors upon interaction with ligands such as 17β-estradiol. Aberrant ER activity is associated with various pathologies including cancer, menopausal syndrome, inflammatory diseases, and others [159]. Tamir et al. [160] screened a panel of natural products from licorice root for estrogenic activity, where 26 (isoliquiritigenin) was found to promote growth of ER positive breast cancer cells T-47D at low concentration whereas at high concentration ER-independent cytotoxicity was observed. Direct binding to ER was studied based on competition with radio labeled ligand 17β-estradiol and 26 revealed low micromolar affinity. This is in agreement with the results obtained by Hajirahimkhan et al. [161] who also confirmed the induction of ER-responsive Tff1 mRNA upon treatment of 26. Similarly, Hegazy et al. [162] isolated several natural products from *Tephrosia candida* and identified 89 (candidachalcone) as a ER ligand with mid-micromolar affinity, although no efficacy data was given.

#### Other receptors

**Figure 18:**
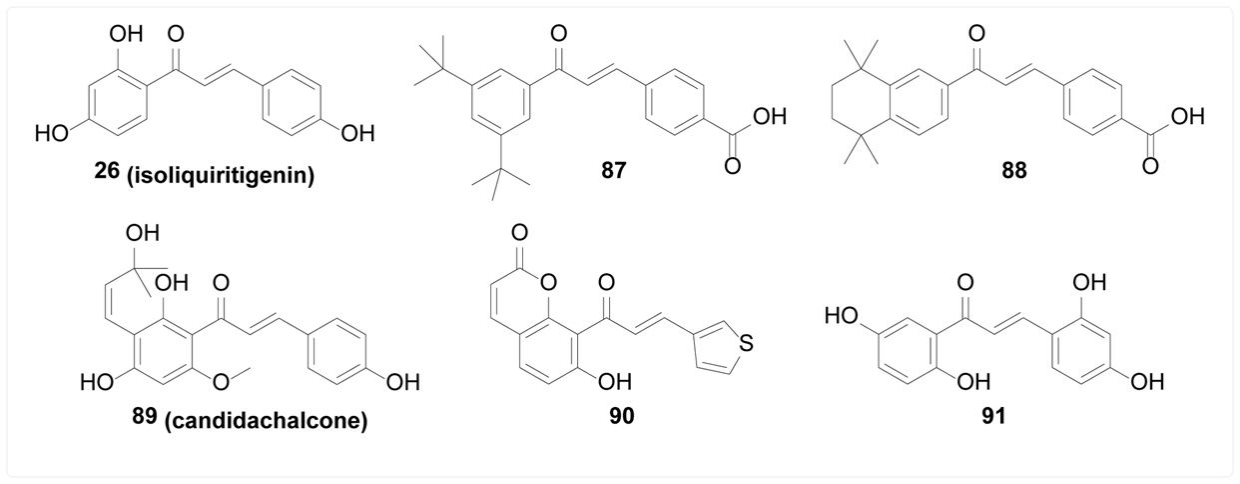
Structures of chalcone compounds as ligands of various receptors.

Compound 26 (isoliquiritigenin) has also been identified as a positive allosteric modulator of GABA_A_ receptor by Cho et al. [163], and a histamine H2 receptor antagonist by Kim et al. [164], potentially explaining its neurological and gastric protective effects. Vazquez-Rodriguez et al. [165] synthesized a panel of coumarin chalcones as potential ligands of adenosine receptors where compound 90 was shown to bind to A_1_, A_2A_, and A_3_ receptors with micromolar potency. Unfortunately, no efficacy data were available. Balsera et al. [166] screened a library of natural products and identified 26 (isoliquiritigenin) as a positive allosteric modulator of α_7_ nicotinic acetylcholine receptor, based on which a panel of chalcones were synthesized and 91 was identified as the most potent compound with *in vitro* neuroprotective effect and *in vivo* analgesic and cognitive enhancing effect (Figure 18).

### Other proteins

#### ABC transporters

**Figure 19:**
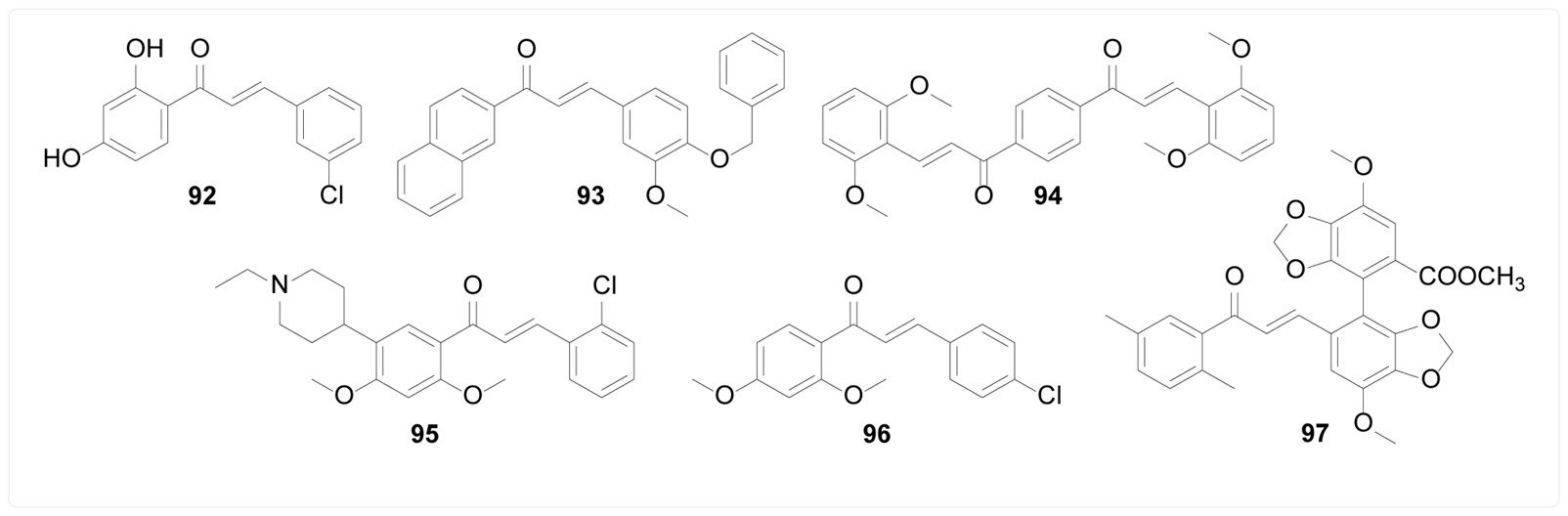
Structures of chalcones as inhibitors of ABC transporters.

ATP-binding cassette (ABC) transporters, such as p-glycoprotein (P-gp) and breast cancer resistant protein (BCRP), are a group of efflux pumps capable of transporting drugs out of the cells, whose overexpression are critical in conferring multi-drug resistance (MDR) in many cancer cell lines [167]. Han et al. [168] synthesized seventeen chalcones, most of which including compound 92 showed BCRP inhibitory activity, increased cellular accumulation of mitoxantrone and sensitized resistant cells to mitoxantrone. None of the chalcones inhibited the ATPase activity of BCRP, suggesting a different interaction mode from canonical ABC inhibitors. Rangel et al. [169] screened fifty-four synthetic chalcones for BCRP inhibition by monitoring cellular mitoxantrone accumulation, and one of the most potent compounds 93 was non-cytotoxic itself but able to sensitize resistant cells to mitoxantrone to a level comparable to the sensitive cells. Similarly, Winter et al. [170] constructed a library of symmetric bis-chalcones and tested their BCRP inhibitory activity with the same method, where activity from zero to complete inhibition was observed with 5 μM of chalcones, demonstrating a sharp SAR relationship. The most potent compound 94 completely sensitized resistant cells to mitoxantrone at sub-micromolar concentration. Mechanistically, 94 alone slightly stimulated basal ATPase activity of BCRP, but completely inhibited the substrate-induced ATPase activity increase. Canonical BCRP inhibitors through ATPase inhibition significantly synergized with 94, suggesting a non-classical binding interaction of chalcones with BCRP, which is consistent with the aforementioned study. Liu et al. [171] synthesized twenty-five structurally diverse chalcones and evaluated their inhibitory effect against both P-gp and BCRP using resistant cells specifically expressing either form. Selective inhibitors were found where compound 95 potently inhibited P-gp but not BCRP and 96 selectively inhibited BCRP over P-gp. Gu et al. [172, 173] designed a panel of bifendate-chalcone hybrid as potential P-gp and BCRP dual inhibitors. The most potent compound 97 inhibited P-gp and BCRP mediated efflux and sensitized resistant cells to adriamycin and mitoxantrone without showing cytotoxicity by itself or inhibiting the ATPase activity of P-gp. The sharp SAR was explored by Parveen et al. [174] with a 3D-QSAR model that gave some structural insight into such interactions (Figure 19).

#### Topoisomerases

**Figure 20:**
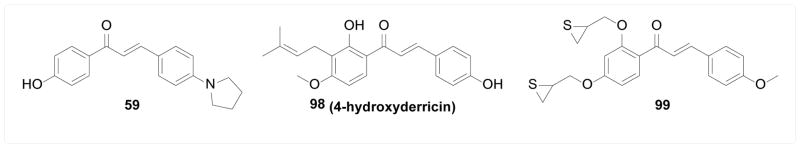
Structures of chalcones as TOPO inhibitors.

Topoisomerases (TOPOs), including TOPO-I and TOPO-II, are enzymes regulating the winding and unwinding of DNA, a critical process for DNA transcription and replication. Therefore, TOPO inhibitors have been widely used as anticancer and antibacterial agents [175]. Several chalcones have shown TOPO inhibitory activity. The aforementioned study by Kim et al. [114] not only identified cytotoxic chalcone 59 as a cathepsin inhibitor, but also a nonselective TOPO-I/II inhibitor with comparable potency as camptothecin and etoposide. However, the contribution of such inhibition to cytotoxicity was not studied. Akihisa et al. [176] studied the cytotoxicity of compounds isolated from *Angelica keiskei* against various cancer cells and the most potent compound 98 (4-hydroxyderricin) were found to inhibit TOPO-II more potently than etoposide, a clinically used anticancer drug acting as a TOPO-II inhibitor. Na et al. [177] obtained several chalcones with selective TOPO-II inhibitory activity over TOPO-I, and the most potent inhibitor 99 also demonstrated the highest cytotoxicity towards a panel of cancer cell lines (Figure 20).

#### MD-2

MD-2 is a small secreted glycoprotein that binds to both Toll-like receptor 4 (TLR4) and LPS, and thereby mediates LPS-induced TLR4 activation which triggers signal transductions related to inflammatory responses [178]. Chalcone compound 100 was identified by Roh et al. [179] via high-throughput screening as an inhibitor of LPS-induced inflammation and mechanistically as a MD-2 ligand. Such binding interaction antagonized LPS binding to MD-2 and led to inhibition of LPS-induced NF-κB activation in cells. Similarly, Wang et al. [180] investigated the anti-inflammatory mechanisms of compound 101, and found that it bound to MD-2 and antagonized the binding of LPS in biochemical assays. In cells, 101 inhibited LPS-induced MAPK phosphorylation, NF-κB activation, and cytokine expression. *In vivo*, 101 enhanced survival and reduced lung injury in LPS-induce septic mice. Moreover, arginine 90 and tyrosine 102 were found to be crucial residues as mutation of either of them abolished the binding of 101 to MD-2, giving insight into its potential binding site.

#### MDM2

As one of the mechanisms by which cancer cells evade apoptosis, overexpression of the oncoprotein MDM2 inhibits tumor suppressor protein p53 by binding to its transactivation domain and thus prevents p53-induced apoptosis of defective cells. Disruption of the interaction between MDM2 and p53 has thus become an interesting approach to combat cancer. Stoll et al. [181] studied the potential of chalcones to disrupt the MDM2/p53 interaction with ELISA and NMR methods, both of which identified compound 102 as the most potent inhibitor of this interaction through binding to MDM2. Resolution of individual residue signals with 2D NMR gave insight into the binding site, which was characterized as the tryptophan pocket defined by S92, V93, L54, G58, Y60, V93, and F91. However, no further biological study was performed to confirm this activity in cells.

#### Cholesteryl ester transfer protein

**Figure 21:**
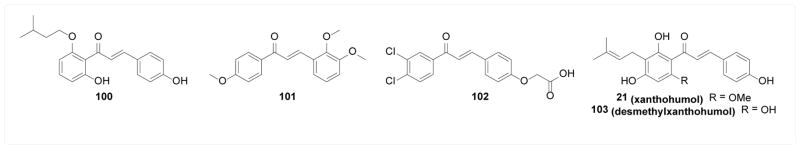
Structures of chalcones acting as MD-2 or MDM2 ligands, or CETP inhibitors.

Cholesteryl ester transfer protein (CETP) is a transporter protein shuttling triglycerides and cholesteryl esters among lipoproteins VLDL, LDL, and HDL; it therefore plays important regulatory roles in lipid metabolism. Modulation of CETP activity is considered a promising strategy to reduce cardiovascular risks [182]. Hirata et al. [183] screened for CETP inhibitory activity from various plant extractions. From the most potent extraction, 21 (xanthohumol) was identified as the major component responsible for this inhibition as a non-competitive inhibitor with mid-micromolar potency. Further SAR study revealed the requirement of the prenyl substituent for potency and identified 103 (desmethylxanthohumol) as a slightly more potent CETP inhibitor (Figure 21).

## Discussion

The diverse bioactivities and target profiles of chalcones is envisioned to be a double-edged sword. On one hand, it gives great opportunities to explore this simple pharmacophore for various target interactions, making it a privileged structure in drug design [184]. On the other hand, it presents the challenge of promiscuity, or poor selectivity, in their biological interactions. Other than in a limited cases where multiple target interactions are synergistic and therapeutically advantageous, such as COX-2/5-LOX dual inhibitors for anti-inflammation [66] and AChE/BACE1 dual inhibitors for anti-AD [185], these multiple-target phenomena would potentially lead to off-target interactions and hence unwanted side effects and compromised potency. The potential promiscuity may explain at least partly why there has been limited clinical success with chalcones in spite of its wide-spread research interest. Therefore, it is of great importance to identify the direct molecular targets responsible for these bioactivities, and ideally characterize their interaction modes, so their target profile can be fine-tuned to achieve better selectivity and potency. As reviewed above, there have been a wide variety of proteins identified as potential direct targets of chalcones, with many chalcone compounds implicated in multiple target interactions (Table 1). Such multi-target profile not only suggests promiscuity of the chalcone template, but also complicates the determination of contribution from each individual target to the overall bioactivity. For example, many targets reviewed here are potentially responsible for chalcones’ cytotoxicity, such as microtubule, Aurora kinases, TrxR, HDACs, and TOPOs, but knowledge of their relative contributions for a given chalcone compound is largely lacking. Those targets showing sharp SAR, such as microtubule and BCRP, are thus of greater interest than those with flat SAR such as 5-LOX and cathepsins, because sharp SAR typically indicates specific binding modes which may be fine-tuned to achieve improved potency and better selectivity.

Special caution is needed when interpreting different levels of evidence for direct target interactions. With *in vivo* or cell-based studies, an observed effect for a target could be due to altered protein level, post-translational modification, direct interaction with compound, or their combinations. For this reason, *in vitro* or *in vivo* evidence alone, without direct biochemical confirmation, is premature to suggest a direct target interaction. On the other hand, although biochemical assays, including X-ray co-crystal structures, provide evidence for direct target interaction, there isn’t a necessary connection to its biological activities without some *in vitro* or *in vivo* experiments, as the compound may preferentially interact with other targets. The result is therefore most convincing when the biochemical studies matches the *in vitro* and *in vivo* activities, which is the case with some of the studies reviewed here, such as some of the anti-microtubule, RTK inhibitory, and ALR2 inhibitory chalcones. On the other hand, the data for some other targets are less convincing and deserve further investigations.

In conclusion, we have attempted to summarize the current knowledge of potential direct molecular targets of chalcone compounds in this review. The wide variety of their potential targets explains at least partly the diverse bioactivities of chalcones, which presents both opportunities and challenges in developing chalcone-based therapeutic agents. A better understanding of the interaction modes and biological relevance of such interactions would greatly facilitate further development of chalcone-based therapeutic agents.

## Figures and Tables

**Table 1:** Examples of multi-targeting chalcones and potential relevance to their diverse bioactivities.

Compound	Potential direct target	Potential bioactivity relevance
Isoliquiritigenin	EGFR	Anticancer
	tyrosinase	Anti-pigmentation
	ALR2	Anti-diabetic, Anti-inflammation
	BACE1	Anti-AD
	ER	Growth Promotion
	GABA_A_ receptor	Anxiolytic/Sedative
	H_2_ receptor	Gastric Protection
	α7-nAChR	Analgesic, Cognitive Enhancing
Butein	IKKβ	Anticancer, Anti-inflammation
	EGFR	Anticancer
	tyrosinase	Anti-pigmentation
	aromatase	Anticancer
	ALR2	Anti-diabetic, Anti-inflammation
	HDAC	Anticancer
Xanthohumol	IKKβ	Anticancer, Anti-inflammation
	COX	Anti-inflammation
	aromatase	Anticancer
	α-glucosidase	Anti-diabetic
	CETP	Anti-dyslipidemia
Licochalcone A	IKKβ	Anticancer, Anti-inflammation
	PI3K, B-Raf, MEK1	Anti-inflammation
	PTP1B	Anti-diabetic
Broussochalcone A	PKC	Anti-inflammation
	COX	Anti-inflammation
	PTP1B	Anti-diabetic
Kuraridin	ALR2	Anti-diabetic, Anti-inflammation
	α-glucosidase	Anti-diabetic
Xanthoangelol	Aurora kinases	Anticancer
	EGFR	Anticancer
